# Environmental impacts on the structural integrity of British rhodoliths

**DOI:** 10.1038/s41598-023-40292-5

**Published:** 2023-08-18

**Authors:** Leanne A. Melbourne, Juliet Brodie, Emily J. Rayfield, Danna Titelboim, Oliver T. Lord, Daniela N. Schmidt

**Affiliations:** 1 School of Earth Sciences, University of Bristol, Bristol, BS8 1RJ UK; 2 Earth and Planetary Sciences, American Museum of Natural History, New York, NY 10024 USA; 3 Department of Life Sciences, Natural History Museum, London, SW7 5BD UK; 4 Department of Earth Sciences, University of Oxford, Oxford, OX1 3AN UK

**Keywords:** Biomaterials, Ecology, Biogeochemistry, Climate sciences, Ocean sciences

## Abstract

Coralline algae form complex habitats which are biodiversity hotspots. Experimental studies suggest that climate change will decrease coralline algal structural integrity. These experiments, however, lack information on local morphological variability and how much structural change would be needed to threaten habitat formation. Here, using finite element modelling, we assess variability in cellular structure and chemical composition of the carbonate skeleton of four coralline algal species from Britain in contemporary and historical specimens collected over the last 130 years. Cellular and mineral properties are highly variable within species, between sites and through time, with structurally weaker cells in the southern species and contemporary material compared to northern taxa and historical material. Yet, temporal differences in strength were smaller than spatial differences. Our work supports long term experiments which show the adaptation potential of this group. Our results suggest that future anthropogenic climate change may lead to loss of habitat complexity in the south and expansion of structurally weaker southern species into northern sites.

## Introduction

Coralline algae are calcified red (Rhodophyta) macroalgae recognised as important habitat-formers in shelf seas in mid to high latitudes^1^. They precipitate high-Mg calcite, the most soluble polymorph of calcium carbonate^2^, within their cell walls, making these organisms vulnerable to ongoing and potential future ocean acidification. Experimental studies have shown negative impacts of warming and increasing CO_2_ conditions on cell size^3^, geochemistry^4^ and molecular bonding^5^. Changes in chemical composition, growth and calcification are important controls on structural integrity and habitat formation, as larger cells and thinner walls lead to weaker skeletons^3,5,6^. Thereby any potential weakening in the structural integrity of the skeleton can lead to increased fragmentation, and result in a loss of the habitat forming ecosystem function.

Rhodoliths, free-living forms of coralline algae, are important habitat formers and protected under EU law (Council Directive 92/43/EEC) with many Special Areas of Conservation (SAC) in the UK focussing on beds generated by rhodoliths. Therefore, changes to the internal structure of rhodoliths has implications for the composition and integrity of the habitats that form. Most studies on the response of coralline algae to elevated CO_2_ have been short term but how an individual organism responds in the long term can be very different^3,7^. Experiments on longer timescales suggest that short term reductions in calcification in response to environmental change in coralline algae can be reversed^7,8^, and therefore highlight the morphological plasticity of coralline algae to respond to and acclimate to changes in the local environment^7,9–11^. Consequently, it has been suggested that coralline algae are more susceptible to rates of ocean acidification than magnitude^5^. Coralline algae have also shown evidence of being able to adapt to tolerate lower pH levels after multi-generational exposure^12^. These combined responses might enable rhodoliths to continue growing under challenging conditions and potentially maintain their function as habitat formers. Hence, long term field studies that incorporate the effects of acclimation and adaptation can provide insight into how structural integrity and habitat function will be affected.

Within any given rhodolith bed multiple coralline algal species can be found, contributing to the habitat formation^13^. As such species-specific responses to climate change could result in extirpation or migration leading to the creation of new niches. In crustose coralline algae, morphology-dependent responses to ocean acidification have led to changes in competitive abilities^14^. Therefore, species-specific responses will need to be accounted for when predicting future responses to climate change and community dynamic changes^15^.

To address questions relating to the potential for acclimation, adaptation, and geographic and species-specific responses, we have used historical records of coralline algal growth over the last century, based on specimens archived in the Natural History Museum, London. Traditionally, historical material has been used to assess changes in distribution and phenology, reconstruct past environments and analyse ecological and evolutionary responses^16–18^. In contrast, morphological responses of organisms are less frequently studied^19^ and structural implications rarely assessed.

**Figure 1:**
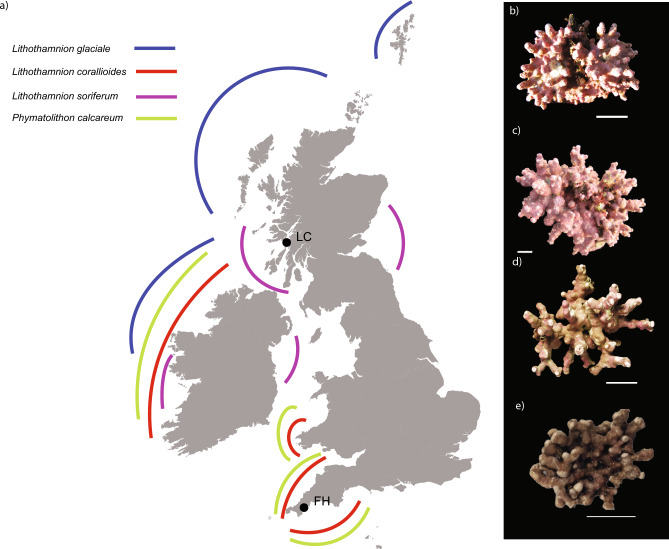
(**a**) A map of the sampling locations with a schematic of the distribution of the main rhodolith builders in the UK (*Phymatolithon calcareum*, *Lithothamnion corallioides* and *L. glaciale*) [adapted after^21^]. *L. soriferum* has been added based on recent studies^13,20^. FH is the site at Falmouth (Fal & Helford SAC), while LC is the site at Oban (Loch Creran SAC). The St Mawes Bank site is within FH. (**b**–**e**) Images of the four species in this study, *L. glaciale* (**b**), *L. soriferum* (**c**), *P. calcareum* (**d**) and *L. corallioides* (**e**), scale bar: 1 cm. Images b and e were adapted from^13^.

Here we test the impact of environmental change over the last 130 years, determine species specific responses and ask whether contemporary specimens are forming weaker skeletons than their historical counterparts. We quantify differences in the cellular structure (cell lumen length and width; interwall and intrawall thickness) and magnesium/calcium (Mg/Ca) ratios of the carbonate skeleton of four species found around Britain (Fig. 1). We compare modern specimens with historical coralline algal collections housed in the Natural History Museum, London (NHM) algal herbarium (BM) collected over the last 130 years from the Fal and Helford area. For regional comparisons, we focus on *Lithothamnion glaciale* and *L. soriferum* (as *L. erinaceum*; see Melbourne, et al.^13^ and Peña, et al.^20^) from the Loch Creran Special Area of Conservation (SAC) in the north of Britain and *L. corallioides* and *Phymatolithon calcareum* from the Fal and Helford SAC in the south of Britain. Theoretical computer models based on the internal cellular structure, representing different species and time periods, were generated to compare environmental impact on stress and strain distributions. From these we infer the effect of environmental change on rhodolith structural integrity and the impact of global change on future rhodolith habitat formation.

## Results

### Understanding natural variability within and between species

Overall, morphology is highly variable within individual taxa, locations and between species with lumen length the most variable cell parameter and interwall thickness the least. For example, intraspecific variability in *Phymatolithon calcareum* cell lumen lengths ranges between 5.37 and 19.61 μm, cell lumen widths between 4.00 and 12.46 μm, intrawall thickness between 0.45 and 3.56 μm and interwall thickness between 0.63 and 4.09 μm. Consequently, individuals within *P. calcareum*, have statistically significant differences in all cell parameters (Length: F_8, 40_ = 7.359, *p* < 0.005; Width: F_8, 40_ = 5.609, *p* < 0.005; Intrawall thickness: F_8, 40_ = 4.2499, *p* < 0.005; Interwall thickness: F_8, 40_ = 2.212, *p* = 0.0469, S1).

**Figure 2:**
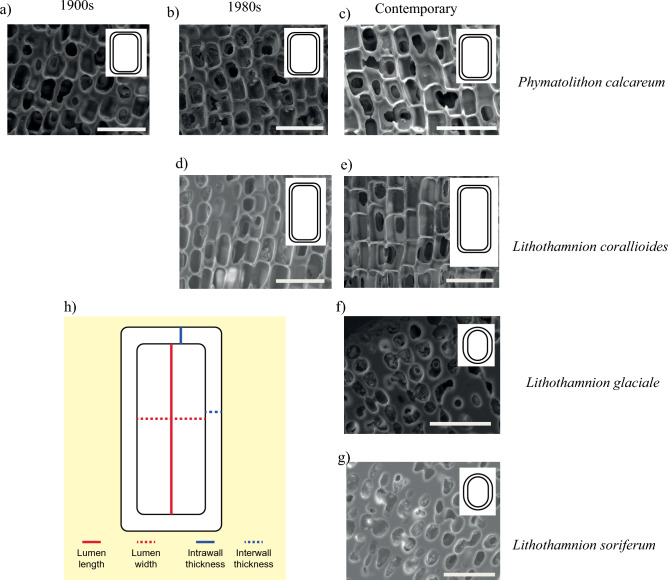
Examples of scanning electron microscopy images for the four coralline algal species *Phymatolithon calcareum* (**a**–**c**), *Lithothamnion corallioides* (**d**–**e**), *L. glaciale* (**f**) and *L. soriferum* (**g**) for each time-period (1900s, 1980s and contemporary) used in this study. Schematic of cell to indicate measurements made (**h**), insets in (**a**–**g**) show stylised cell shape for each species and time-period. Scale bar: 30 µm.

These differences of cellular shape and dimensions (Table 1) are larger between regions and smaller between species at the same location, highlighting the combined importance of taxonomy and environment. Southern taxa *P. calcareum* and *L. corallioides* have larger and more rectangular cells in comparison to *L. glaciale* and *L. soriferum* which have rounder, smaller cells (Table 1, Fig. 2). Both lumen length and width in *L. glaciale* and *L. soriferum* cells are significantly smaller than those for *P. calcareum* and *L. corallioides* (length: X^2^ = 58.368, d.f. = 3, *p* < 0.005; width: X^2^ = 61.055, d.f. = 3, *p* < 0.005). At the same location, cell lumen is longer in *L. corallioides* than in *P. calcareum* (X^2^ = 58.368, d.f. = 3, *p* < 0.005), whereas there are no differences between *L. glaciale* and *L. soriferum*. Cell lumen widths at each locality are the same (Table 1). *L. corallioides* has significantly thinner intrawalls than *L. glaciale* and *P. calcareum* (X^2^ = 22.427, d.f. = 3, *p* < 0.005); *L. corallioides* and *P. calcareum* have significantly thinner interwalls than *L. glaciale* and *L. soriferum* (X^2^ = 22.04, d.f. = 3, *p* < 0.005).

**Table 1:** Summary of the morphological and geochemical data of the contemporary specimens from Oban (*L. soriferum* and *L. glaciale*) and Falmouth (*L. corallioides* and *P. calcareum*).

	*Lithothamnion soriferum*	*Lithothamnion glaciale*	*Lithothamnion corallioides*	*Phymatolithon calcareum*	Statistics
Lumen length (µm)	8.92 ± 0.13 (A)	8.88 ± 0.09 (A)	18.49 ± 0.25 (C)	12.0 ± 0.06 (B)	**
Lumen width (µm)	6.11 ± 0.08 (A)	5.82 ± 0.07 (A)	8.14 ± 0.05 (B)	7.79 ± 0.04 (B)	**
Intra-cell wall (µm)	1.76 ± 0.06 (AB)	1.86 ± 0.04 (B)	1.41 ± 0.02 (C)	1.49 ± 0.01 (AC)	**
Inter-cell wall (µm)	2.44 ± 0.09 (A)	2.35 ± 0.05 (A)	1.66 ± 0.02 (B)	1.72 ± 0.01 (B)	**
Mg/Ca ratio (mol/mol)	0.186 ± 0.0002 (A)	0.202 ± 0.0003 (AB)	0.228 ± 0.0004 (B)	0.180 ± 0.0003 (A)	**

Numbers are mean ± SE, standard error of the mean. ** represents results that have a *p*-value below 0.005, letters represent the different statistical groups.

**Figure 3:**
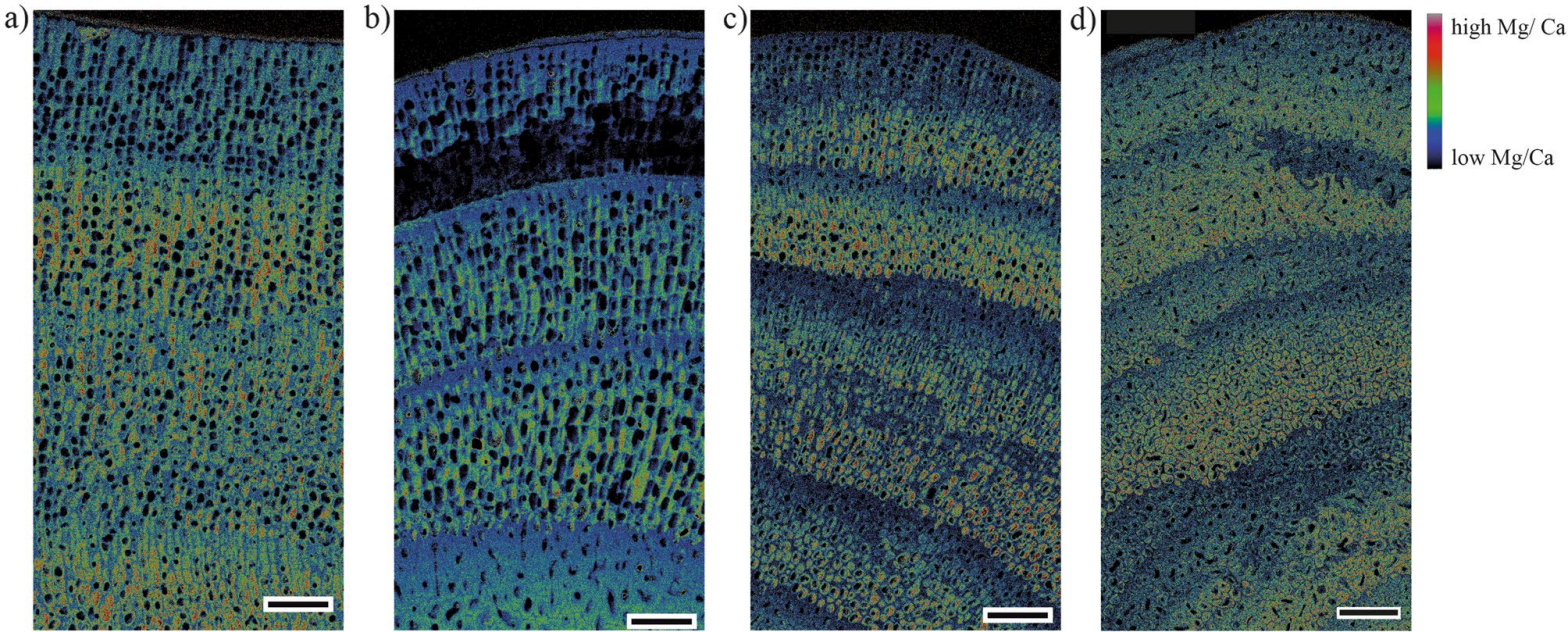
Microprobe Mg/Ca maps of (**a**) *Phymatolithon calcareum*, Falmouth, UK, 2014, (**b**) *Lithothamnion corallioides*, Falmouth, UK, 2014, (**c**) *L. glaciale*, Oban, UK, 2014, and (**d**) *L. soriferum*, Oban, UK, 2014. Scale bar: 50 µm.

As expected, Mg/Ca ratios differ between species but are also highly variable within an individual. Mg/Ca ratios in *L. soriferum* vary between branches and individuals (0.172 to 0.213 mol/mol) (F_2, 13476_ = 459.6, *p* < 0.005, S1). *L. corallioides* has significantly higher Mg/Ca ratios than *P. calcareum* and *L. soriferum* (X^2^ = 11.905, d.f. = 3, *p* < 0.005; Table 1). *L. corallioides*, *L. glaciale* and *L. soriferum* show a strong seasonal periodicity in the Mg/Ca maps, while the growth bands are not as distinct in *P. calcareum*, but still visible (Fig. 3). Using the temperature equations from Kamenos, et al.^22^, all species show similar annual temperature ranges (4 °C to 16.5 °C), and therefore we could not resolve the small differences in the temperature ranges between the two sites.

### Historic versus contemporary: comparing skeletal change through time

Cell sizes in contemporary specimens are generally larger than in historical material. For *P. calcareum* and *L. corallioides* contemporary specimens have thicker cell walls than material from the 1980s. For *P. calcareum* only the intrawalls are thicker in the contemporary material compared to the 1900s material and the interwall is thicker in the 1900s material compared to contemporary material (Table 2, Fig. 2). However, none of the differences in cell parameters are statistically significant (Table 2).

**Table 2:** Summary of the comparison of morphological and geochemical data for historical and contemporary coralline algae from Falmouth (*L. corallioides* and *P. calcareum*).

	*Phymatolithon calcareum*			*Lithothamnion corallioides*		Statistics
	1900s	1980s	2014	1980s	2014	
Lumen length (µm)	11.15 ± 0.14	11.36 ± 0.02	12.01 ± 0.06	16.12 ± 0.32	18.49 ± 0.15	–
Lumen width (µm)	7.35 ± 0.12	7.81 ± 0.12	7.79 ± 0.04	7.61 ± 0.13	8.14 ± 0.05	–
Intracell wall (µm)	1.23 ± 0.05	1.18 ± 0.04	1.49 ± 0.01	1.39 ± 0.04	1.41 ± 0.02	–
Intercell wall (µm)	1.83 ± 0.05	1.65 ± 0.05	1.72 ± 0.01	1.57 ± 0.04	1.66 ± 0.02	–
Mg/Ca ratio (mol/mol)	0.348 ± 0.001 (A)	0.262 ± 0.001 (A)	0.180 ± 0.0003 (B)	0.399 ± 0.001 (A)	0.228 ± 0.0004 (B)	**

Numbers are mean ± SE, standard error of the mean. ** represents results that have a *p*-value below 0.005 and / represents results that are not statistically significant, letters represent the different statistical groups within each morphological parameter.

Mg/Ca ratios in historical samples are higher than in contemporary specimens (X^2^ = 31.993, d.f. = 2, *p* < 0.05) (Table 2) but there are no significant differences between the early 1900s and mid 1980s. Temperatures determined from Mg/Ca values for the historical material range from 8 °C to 22 °C for *P. calcareum* and 9 °C to 28 °C for *L. corallioides* indicating temperatures which are too high for the region.

### Characterising the mineralogy

**Figure 4:**
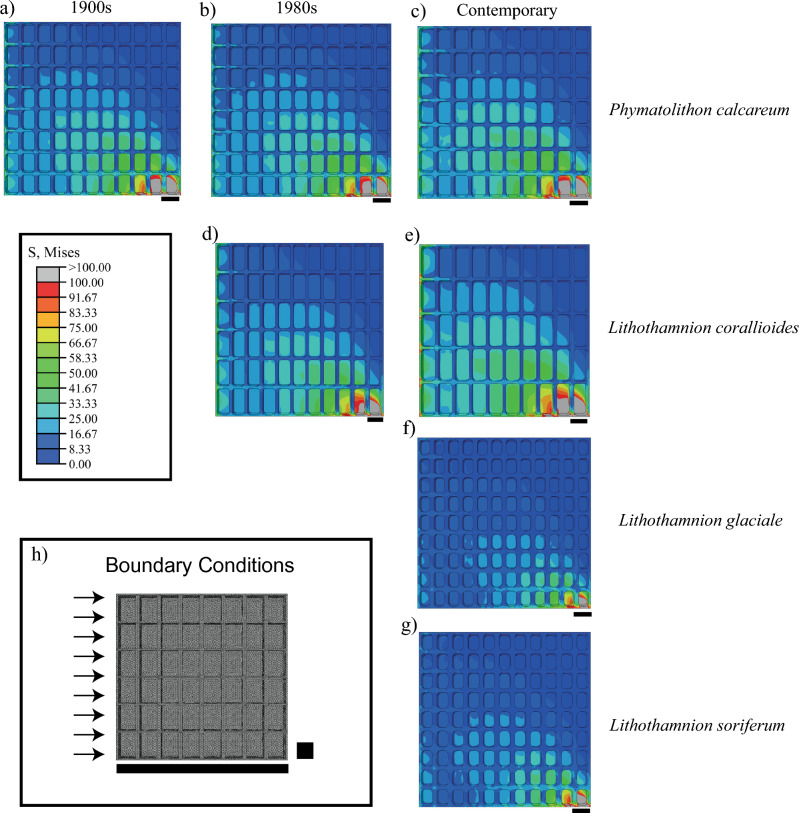
Finite element models showing the differences in stress distribution associated with the morphology of the investigated species through time under shear loading (**a**–**g**). Red colours indicate high stress and blue colours lower stress. The small line in the bottom right corner of the model indicates where load was applied. Schematic of the boundary conditions where the arrows represent the loads and the black box represents the constraints applied (**h**). Scale bar: 10 μm. Units: MPa.

Given the current discussion around the mineralogy of coralline algae, how it may respond to future warming and its importance for material properties, we performed Raman spectroscopy on a polished section of a sample of *Lithothamnion corallioides* from the south of Britain collected in 1982 (HCor82). This sample was chosen due to the high Mg/Ca values measured by Electron Probe microscopy (EPMA) and therefore we wanted to see if these values correlated with a change in mineralogy, e.g., an incorporation of dolomite into the skeleton. The positions of all four peaks in the Raman spectra fall approximately half-way between the values expected for pure calcite and dolomite. Using the Borromeo, et al.^23^ calibration for biogenic Mg carbonates, this translates to a MgCO_3_ content of between 32.6 and 43.3 mol%, which is lower than values derived by EPMA for the same sample (50 mol%). The ν1 mode has a distinct asymmetry, with a tail extending to low wavenumbers. While this is a feature of dolomite, it is also present in magnesian calcite with MgCO_3_ contents as low as 25 mol% (see e.g. Figure 4d of Perrin, et al.^24^). The ν2 mode at 880 cm^−1^ that is indicative of dolomitic ordering (though not necessarily dolomitic stoichiometry) is not observed, which is not uncommon for this exceptionally weak mode. The only spectral feature, other than the peak positions of the T, L, ν1, and ν4 modes that can provide definitive proof of the presence of stoichiometric dolomite, is a peak at 340 cm^−1^ that does not appear in magnesian calcite, even with a degree of dolomitic ordering. While there is a small feature at this position in our spectra, it is not statistically significant at the 95% confidence level, and the presence of additional peaks at near regular spacings (e.g., 390 cm^−1^, 440 cm^−1^ and 500 cm^−1^) suggest that this is probably an interference fringe. In summary, while the data do not preclude the presence of dolomite, they can be adequately explained by high magnesium calcite, with no need for an additional mineral phase.

### Structural implications on growth

Larger cells with thinner walls result in higher magnitude stresses and strains for the finite element models representing *P. calcareum* and *L. corallioides* compared to *L. soriferum* and *L. glaciale* (Fig. 4). *L. corallioides* display the highest von Mises stress and *L. glaciale* the lowest von Mises stress and strain energy. The pattern of stress distribution within the skeleton is broadly similar amongst species but higher magnitude stresses extend further through the structure in *P*. *calcareum* and *L*. *corallioides* (Fig. 4, Table 3, S2 for images of stress distribution under compression loading).

**Table 3:** Summary table of the stress distribution in the contemporary and historical specimen, picojoules (pJ) and megapascals (MPa).

Species	Year	Loading	# of elements	Strain energy (pJ)	Mean von Mises stress (MPa)	95th percentile von Mises Stress (MPa)	95th percentile Max Principal stress (MPa)
*Phymatolithon calcareum*	1900s	Compression	2,673,700	667.33	7.51	9.66	2.24
	1980s		3,243,061	654.71	7.03	9.28	2.17
	Contemporary		2,686,121	707.22	8.21	10.78	2.40
*Lithothamnion corallioides*	1980s		3,506,921	671.04	7.16	8.99	2.12
	Contemporary		2,511,080	768.67	8.72	10.97	2.63
*Lithothamnion soriferum*	Contemporary		3,843,995	401.63	5.81	7.25	1.50
*Lithothamnion glaciale*	Contemporary		4,509,259	365.39	5.25	6.59	1.38
*Phymatolithon calcareum*	1900s	Shear	2,673,700	4623.5	13.64	34.67	11.69
	1980s		3,243,061	4583.86	12.96	32.95	10.82
	Contemporary		2,686,121	4658.38	14.74	37.66	13.20
*Lithothamnion corallioides*	1980s		3,506,921	4464.5	12.73	36.62	11.41
	Contemporary		2,511,080	5167.58	15.73	40.94	13.82
*Lithothamnion soriferum*	Contemporary		3,843,995	2557.95	10.07	24.36	9.14
*Lithothamnion glaciale*	Contemporary		4,509,259	2391.77	9.13	22.15	7.98

Over time, cell lumens get longer and wider with slightly thicker walls from the 1980s onwards. Overall, this results in an increase in stress and strain energy from historical to contemporary specimens (Table 3, Fig. 4, S2 for images of stress distribution under compression loading). For *P. calcareum* the 95th percentile of the maximal principal stress under shear loading increases by 13% between the 1900s and contemporary material and 22% between the 1980s and the contemporary material, while under compression loading it increases by 7 and 11% respectively. For *L. corallioides* the 95th percentile of maximum principal stress increases by 21% under shear loading, and 24% under compression (S2).

## Discussion

The loss of important habitat forming species is of increasing concern due to the implications for biodiversity and carbon regulation of these habitats^25,26^. However, the predictability of the impacts of climate change is challenging, given that drivers of distribution changes and traits are not sufficiently understood at the species level. Too often presence and absence or physiological performance is assessed, but the structural integrity of species is frequently overlooked^25,27^.

Our study has highlighted the structural variability within rhodoliths spatially and through time and how this variability in growth affects structural integrity and ecosystem function. Crucially, engineering models of species that currently inhabit the south of Britain are structurally weaker than northern species under the same simulated loads. Based solely on differences in morphology, this implies that southern species are more prone to breakage with increased wave action and brings into question the ability of southern species to migrate northwards as the climate changes and the seas warm, if these structural differences are exclusively taxonomically driven^28,29^.

### Variable growth in a highly variable environment

The cellular ultrastructure and mineral chemistry within branches and individuals within a species at one location were highly variable. Given the dynamic environment in shallow waters with diurnal, tidal and seasonal changes and the knowledge of environmental impacts on rhodolith morphology^10,30,31^, this high skeletal variability was expected, confirming previous studies^29^. To account for the variability, we emphasize the importance of using multiple branches and individuals to represent a population.

Despite the large intra-population variability, there are distinct regional differences in cell size and shape, despite overlap in interwall thickness between *P. calcareum*, and *L. soriferum* from the north of Britain. *P. calcareum* is widespread throughout the north Atlantic from the Azores up to Norway and Sweden, unlike *L. corallioides,* whose northern limit reaches Ireland^21,32^. Therefore, the wide geographic range may lead to higher morphological plasticity which may be the reason similar wall thicknesses are found between *P. calcareum* and *L. soriferum*^21^. While there is evidence that species morphology can change along a latitudinal gradient^31^ further work is needed to understand the geographic drivers of internal structure within taxa and the link between niche width and structural plasticity.

Cellular ultrastructure does not show strong trends through time, raising questions relating to the relative importance of external factors (e.g., temperature, light, carbonate chemistry) compared to internal (biological) control. The Western Channel Observatory, the closest long term monitoring station to Falmouth, has recorded warming and increased CO_2_ levels from the 1980s onwards with the end of the twentieth century being 0.5 °C warmer than the first half^33,34^. However, the lack of statistical significance in the cellular structure differences between the historical and modern material suggests that this warming may not be sufficient to impact the internal structure. More specifically, the positive impact of higher CO_2_ on growth might be counterbalanced by a reduction of growth due to heat stress for species at the edge of their distribution^35^, increased metabolic demands in response to warming or the combined impacts of warming and acidification^36^.

Another theory is that the local conditions are masking the effects of warming and acidification. For example, changes in turbidity, which alter the clarity of the water and hence light for photosynthesis^10^, or as warmer, wetter weather increases riverine inputs leading to more nutrients and higher alkalinity^37^, could influence growth and calcification^38^. Furthermore, changes in land use have altered sediment loads in river systems^39^. Changes in legislation in 1992 have led to reductions in nutrient loads, especially phosphate, in rivers^40^, which could potentially decrease productivity in the Fal estuary and light attenuation^38^ supporting growth. Our results therefore highlight the real challenges that come with analysing museum collections, such as small sample sizes and lack of contemporary local environmental information. Without a local record of carbonate chemistry, it is impossible to delineate these contemporaneous and interacting impacts on cellular growth. Given the lack of baseline data on ecological status, these limitations clearly are outweighed by the benefits of generating baseline morphological information where these were missing, especially in the context of marine protection for our sampling locations.

Recent studies on coralline algae have found morphological features that are distinct to species and families^41^, thereby aiding identification. For example, the thickness of the secondary wall in perithallial cells can be used to distinguish between *Lithophyllum racemus* and the cryptic species *L. pseudoracemus*^28^. Even though a strong biological control on some internal features was also shown by Bracchi, et al.^29^, cell length in *Lithothamnion corallioides* decreased with increasing depth highlighting the impact of the local environment on certain parameters. Therefore, a wider range of analysis of contemporary and historical material along a latitudinal gradient would strongly complement our analysis and bring further insights.

### Mineral chemistry is not clear cut

A link between Mg concentration and resistance to deformation has been identified in sea urchin spines (Ma et al. 2008). Therefore understanding differences in elemental distribution across taxa and through time is paramount to our study. Unlike cellular shape, geochemical composition does not change with location^22,42^. The temperature ranges between the two sites are similar (a difference of 3 °C in the minimum and maximum temperatures), which could explain the similar Mg/Ca at the two locations. Additionally, recent research on calcification in corallines suggest that the changes in Mg concentration can be overridden by anatomical changes, which are driven by changes in light (Sletten et al. 2017, Nash & Adey 2018, Nash et al. 2019). Therefore, the interaction of temperature and light induced anatomical changes might obscure such small changes related to temperature.

Our temporal Mg/Ca data taken at face value would be interpreted as cooling, contradicting the warming seen over the last century in the Western Channel^33^. Such a mismatch of geochemical composition and long-term temperature changes was also seen in the articulated coralline species *Corallina officinalis*. Specimens ranging from 1837 to 2010 did not demonstrate expected increases in Mg in the skeleton^43^. We, however, rule out preservation issues as our SEM data did not show any evidence of recrystallisation or partial dissolution. Furthermore, as high Mg-calcite is more susceptible to dissolution we would expect to see lower Mg values if diagenesis occurred.

A change in mineralogy could explain our temporal results as our very high Mg/Ca data may be interpreted as the presence of dolomite, which has been previously found in *Hydrolithon onkodes*^44^ and *Porolithon onkodes*^45^, or the presence of ‘D-type’ carbonate^46^. However, our Raman data does not support this interpretation. No evidence for the presence of dolomite was observed, replicating results in other studies on rhodoliths^47^.

Additionally, other studies have shown that increasing CO_2_ concentrations have led to lower Mg concentration in corallines^4,48,49^ and therefore there may be an impact of carbonate chemistry on Mg concentration which we cannot prove as we do not have an accurate record of the carbonate chemistry at the studied locations. However, our results do raise concerns about the application of the Mg-temperature proxy in the past where structural changes, temperature and local conditions might have been altered differently.

Decreasing Mg content leads to a lower resistance to applied stress in many other taxa^50–52^. In articulated corallines, it has been suggested that the alternating Mg bands within the cells aid in preventing fracture caused by crack propagation^53^. While the link between Mg and material properties has yet to be proven in rhodoliths, it has been suggested that changes to the crystal structure and grain size in corallines have a larger impact on material properties than changing Mg concentration^54^ and that overall structure is more important than geochemical compositions^6^. Therefore, these minor changes in Mg concentration are not likely to have a large negative effect on structural integrity.

### Structural integrity between locations and through time

Our results show that species from the south of Britain are more prone to fracture than species from the north of Britain. We attribute the difference in structural integrity to the larger cells with thinner cell walls leading to less calcite deposited within the skeleton^3^, as previous work has shown that structure impacts structural integrity more than material properties^6^.

Small increases in cell lumen length and width through time led to a slight decrease in structural integrity, though differences in structural integrity were greater between contemporary species. This highlights that long term changes (1900s to present day) in warming and acidification are not yet drastically reducing structural integrity. Our results show the importance of wall thickness in structural integrity, as there are larger differences in structural integrity between contemporary species with distinct differences in wall thickness, compared to the historical material where the differences in wall thickness are much smaller. However, future climate change and associated changes to the chemistry of the coastal waters are suggested to negatively impact calcification^36^, by thinning cell walls^3^, and reducing skeletal density^55^. This projected environmental change may be the catalyst that reduces structural integrity to the point where habitat function is impacted. Under future stormier conditions where increased wave speeds would lead to more intense physical pressure on these organisms^54^, our research emphasises greater vulnerability to climate change of the southern species in Britain than their northern counterparts, which in turn will alter ecosystem services.

### Impact on conservation management

Fundamentally these results highlight the importance in understanding species-specific differences in structural integrity from a conservation perspective. Under increased warming, coralline algae will migrate northward following their temperature optima^56^, as seen in other seaweeds^57^. This migration will alter the composition at a given locality^25^. As marine protected areas are static in nature due to the legal frameworks of their designation in the UK, any migration of taxa out of the defined protected area would challenge their future^58^. Given that the southern species are forming weaker skeletons, we postulate that they are more prone to breakage than the species from the north of Britain. This increased breakage potential combines with projections of increasing sea level and changes to weather patterns, which will lead to larger coastal risk (flooding and coastal erosion) in the south of the UK, compared to the west coast of Scotland^59^. Therefore, we may see increased fragmentation, which may be good for vegetative reproduction^32^, but will increase susceptibility to smothering by silt and reduce habitat generation^10,60^. Furthermore, studies have suggested we may lose rhodolith beds from the northern latitudes due to ocean acidification^56^, and niche availability^25^ leading to range contractions. Most importantly, increased fragmentation will reduce habitat complexity and hence one of the ecosystem functions^61^, threatening protection in SACs^62^. These environmental stressors combined with non-climatic drivers, climate adaptation and mitigation actions in shallow marine habitats, are challenging the future of coralline algal based Marine protected areas^58^.

## Conclusions

Our results demonstrate the need to assess impacts of climate change on internal skeletal morphology given location and species-specific differences. Internal growth is highly variable resulting in significant species-specific differences in internal stress. Our new understanding of the structural vulnerability of rhodoliths calls for multiple stressor experiments or field studies, assessing the impact of nutrients, sediment input, or alkalinity in conjunction with temperature, to assess if management options might alleviate non climatic drivers on growth to ensure the future function of rhodolith beds in marine protected areas.

While externally these coralline algae look similar, the ability to withstand physical pressures is different. Species of coralline algae from the south of Britain are more likely to break under wave exposure than species from the north. Species from the south of Britain could move northwards following their climatic niche with increasing warming. These taxa though will be less able to withstand predicted stormier conditions due to their cellular structure. Such an observation is important given the increasing interest in translocation of species for environmental adaptation. The potential inability of migrating species to replace incumbent species under warmer conditions may lead to changes in rhodolith beds, such as increasing coralline gravels and smaller rhodoliths leading to decreased physical complexity of the ecosystem.

## Materials and methods

### Collections

Specimens from St Mawes Bank, Falmouth, Cornwall, of *Lithothamnion corallioides* (n = 7) and *Phymatolithon calcareum* (n = 9), (mean depth 6 m; July 2014) and from Loch Creran, Oban, Scotland, of *L. glaciale* (n = 6) and *L. soriferum* (n = 3) (depth 9.6 m; October 2014) were hand-collected by scuba divers (Fig. 1). Historical specimens of *L. corallioides* (n = 3) and *P. calcareum* (n = 5) were sampled from the BM algal herbarium at the Natural History Museum, London (S3). All specimens were air-dried without chemical fixation. The specimens were grouped depending on the collection date with historical material covering 1895–1907 (1900s) and 1976–1985 (1980s), and contemporary material (Contemporary) collected in 2014.

Falmouth experiences a maritime climate due to the Gulf Stream. Sea surface temperatures range from 9 °C in winter to 16 °C in summer^33^. Loch Creran is located north of Oban where sea surface temperatures range from 6 °C in winter to 15 °C in summer^63^. All specimens were identified by DNA barcoding using the mitochondrial cytochrome oxidase gene 1, CO1-5P, and the plastid gene, psbA; details can be found in Melbourne, et al.^13^.

### Skeletal analysis: Scanning Electron Microscopy (SEM)

Details of specimens used for each analysis can be found in the supplementary material S3. All samples were clean and free from epiphytes prior to analysis. Individual branches were embedded in epoxy resin and polished to expose the internal thallus structure. Images of cellular structure were taken using a Hitachi S-3500N variable pressure microscope. Cross sections showed bands of larger cells with thinner cell walls and bands of smaller cells with thicker cell walls, representing summer and winter growth respectively^64^. Cell lumen length, width and wall thickness were measured on intact cells within summer growth bands, as these are more regular than cells within winter growth bands and the more open structure will be more important in generating breakage patterns. The amount of winter growth was a lot smaller than the summer growth within a branch and it was assumed that the main contributor to the structural integrity would be the summer growth. Using Image J^65^. 25 ± 10 cells were measured per branch resulting in between 134 and 1225 measurements for each species in each time-period, depending on the number of branches and number of specimens (Table 4, S3).

**Table 4:** Number of individuals and branches (skeletal variability) assessed for each species and time-period.

Species	Time Period	Skeletal variability	Skeletal variability	Mineral variability
		Number of Individuals	Number of branches per individual	Number of Individuals
*L. soriferum*	Contemporary	3	3	3
*L. glaciale*	Contemporary	6	3	6
*L. corallioides*	Contemporary	7	3–5	6
*L. corallioides*	1980s	3	2–3	3
*P. calcareum*	Contemporary	9	5–8	6
*P. calcareum*	1980s	2	2–3	2
*P. calcareum*	1900s	3	1–2	3

A full list of individual specimens can be found in the supplementary material S3.

Contemporary specimens of *Phymatolithon calcareum* were used to assess intraspecies variability. Cell lumen length was chosen as a proxy for variability, as it had the most variability within an individual specimen. It was calculated that over 400 measurements of cell length were required to stabilise around the true mean for each species (S4 & S5). However, due to small numbers of specimens in the historical material and for contemporary *L. soriferum*, a smaller number of measurements (between 130 and 200) was made for each parameter. All groups, however, had a standard error lower than 0.35, with the contemporary species having a standard error below 0.1, indicating higher variability in the historical material (S5).

### Geochemical analysis: Electron Probe Microanalyzer (EPMA)

Measurements of Calcium (Ca) and Magnesium (Mg) were carried out on a JEOL JXA8530F Hyperprobe with an accelerating voltage of 10 kV, a beam current of 40 nA and a dwell time of 5 ms. A defocused beam with a diameter of 0 µm was used and the pixel size of the maps generated was 0.26 × 0.26 µm. Using the Golden Surfer Software (version 12.8) and the CalcImage Probe Software (version 11.8), counts were converted to maps in atomic percent. In ImageJ^65^, Mg maps were divided by Ca maps to generate Mg/Ca molar ratios (mol/mol). Line transects (500 µm in length, 0.26 µm apart) on the map were created from the tip of the thallus down the branch along the midline. Temperatures based on Mg/Ca were calculated using the equations in Kamenos et al.^22^ for the species *P. calcareum* (in situ temperature (IST) = 42.12 [Mg/Ca] + 0.74) and *L. glaciale* (IST = 49.40 [Mg/Ca] − 0.92). As species specific calibrations do not exist for all species, the temperature equation for *P. calcareum* was used for the species at the southern site, while the temperature equation for *L. glaciale* was used for the species at the northern site.

### Mineralogical analysis: Raman Spectroscopy

Raman spectra were collected to investigate the presence of dolomite using a confocal Thermo Scientific DXRxi Raman microscope with a 780 nm laser source operating at 100% power (28 mW), and a 100 × objective yielding a diffraction limited focal volume with a diameter of ∼4 μm. Hyperspectral maps were created using a step size of 4 μm, with the spectrum at each point generated by averaging 5 accumulations each with an exposure time of 1 s. Spectral resolution is ~ 5 cm^−1^. Individual spectra selected from across these maps as well as spectra generated by averaging regions of interest were inspected and compared to synthetic carbonates that range in concentration of the MgCO_3_ component from pure calcite (0 mol% MgCO_3_) to pure dolomite (50 mol% MgCO_3_) as described in Perrin, et al.^24^. Raman maps were collected from both winter and summer bands. A typical spectrum (S6) contains four peaks that can be clearly distinguished from the background, all of which can be indexed to high magnesian calcite, at 166 cm^−1^ (T mode), 288 cm^−1^ (L mode), 720 cm^−1^ (ν4 mode) and 1092 cm^−1^ (ν1 mode).

### Finite element analysis (FEA)

Finite element models were created to quantify the structural implications of changes to growth and to be able to extrapolate to potential loss in ecosystem function. FEA models assessed the response between contemporary species and historical specimens to wave action loading (see Melbourne, et al.^6^ for details). Models for contemporary *Lithothamnion corallioides*, *L. soriferum*, *L. glaciale* and *Phymatolithon calcareum* and the historical material (*P. calcareum* 1900s, 1980s, and *L. corallioides* 1980s) were generated. All models were based on a 100 µm cube to eliminate effects relating to model size and focus on the mechanical effects of internal structure. Each cube was custom constructed according to the cellular sizes measured via SEM. The stylised models were designed to represent a cube of structure in the rhodolith branch^6^. The part of the model where the loads are applied represents the external surface of the rhodolith branch, while the part of the model which is constrained represents the attachment to the rest of the rhodolith branch.

The main hydrodynamic force exerted on marine macroalgae is drag, which occurs in the direction of flow^66^. Due to the position of the cube within the rhodolith and its small size, variation in flow and therefore drag force due to boundary layer effects is minimal. Two loading types were applied to both models: compression and shear. As rhodoliths are unattached to the seafloor both loads can act on the skeleton depending on the orientation of the rhodolith. Our shear model represents the effect of a drag force perpendicular to the long axis of the rhodolith cell, while 'compression' represents a drag force aligned with the long axis of the rhodolith cell. The load applied represented a drag force of 0.03 N, characteristic of a wave velocity of 0.40 m s^−1^ (experimentally determined in Melbourne, et al.^54^). Under compression loading, 18,180 elements were loaded, while 9282 nodes were constrained to prevent translation in the y-direction. Under shear loading, 17,820 elements were loaded, while 9464 nodes were constrained with three degrees of freedom. 9282 nodes were constrained to prevent translation in the y-direction while 182 nodes were constrained to prevent translation in the x-direction. The additional constraint applied under shear loading on the opposite face of the load enabled the model to be computed.

Von Mises stress, strain energy and maximum principal stress within the structure were recorded as in Melbourne, et al.^54^. Von Mises stress is a good predictor of failure in ductile and brittle biological structures^3,6,67^. Total strain energy refers to the energy stored in the system, where more strain energy leads to a larger potential for fracture^68^. When maximum principal stress exceeds the maximum yield strength of the material in tension is a good predictor of fracture in brittle materials. Therefore, higher stress and strain values indicate models that are more prone to failure and fracture. The 95th percentile for von Mises and maximum principal stress was reported to exclude the extreme erroneous stresses caused by elements adjacent to the loads and constraints.

All 3D geometric models were created and analysed in the Finite Element software package, Abaqus/CAE, v.6.14, (Simula, USA, Dassault Systémes, //Simula, Providence, RI, USA) using the Young’s modulus of the summer growth of *P. calcareum* (E = 19.37 Gigapascal (GPa) used for *P. calcareum* and *L. corallioides*) and the summer growth of *L. glaciale* (E = 23.02 GPa, used for *L. glaciale* and *L. soriferum*)^54^. The Young’s modulus represents the stiffness of the material, i.e., the tensile stress over the tensile strain. The Young’s modulus of only one species at each site was used as it was shown in Melbourne, et al.^6^ that a larger difference in the Young’s modulus (larger than what is seen between the two sites) did not impact the von Mises stress in our FEA models.

### Statistics

Statistics were calculated in RStudio (version 4.1.1). A nested ANOVA was used to assess intraspecies variability between individuals and branches. A least squares Tukey model was performed as a post hoc test. The data fits a normal distribution and was determined to be unbiased and homoscedastic. For assessing interspecies and temporal variability a mixed effects model was used with species and time as fixed effects and individuals classed as random effects. A modified Tukey model was performed as a post hoc test. For the full code see the supplementary material (S7). Within a morphological parameter, groups that share the same letter are not statistically different from each other.

He Ethics.

Sampling consent of the protected species was given by Natural England and Marine Scotland. All specimens are in the BM algal herbarium at the NHM.

### Supplementary Information

Supplementary Information.

## Data Availability

The data underlying this article are available in Pangaea data archive at https://doi.org/10.1594/PANGAEA.899694 and at the University of Bristol data repository, data.bris, at 10.5523/bris.1zi10bsu9kxpa1z7w1eoy43wcf.
