# Supplementary material for: Environmental impacts on the structural integrity of British rhodoliths
Source: Sci Rep. 2023 Aug 18;13:13473. doi: 10.1038/s41598-023-40292-5 (PMC10439216; doi:10.1038/s41598-023-40292-5)
Supplement: Supplementary file 1 — Supplementary Information. [file 41598_2023_40292_MOESM1_ESM.pdf]

Supplementary Information

Environmental impacts on the structural integrity of British rhodoliths

Authors: Leanne A. Melbourne[1,2]*, Juliet Brodie[3], Emily J. Rayfield[1], Danna Titelboim[1,4], Oliver T. Lord[1] and Daniela N. Schmidt[1],

[1]School of Earth Sciences, University of Bristol, Bristol, BS8 1RJ, UK,

[2] Earth and Planetary Sciences, American Museum of Natural History, New York, NY 10024

[3] Department of Life Sciences, Natural History Museum, London, SW7 5BD, UK

[4] Department of Earth Sciences, University of Oxford, Oxford, OX1 3AN, UK

S1: Table of morphological characters within *Phymatolithon calcareum* and the geochemical variability within *Lithothamnion glaciale*. SE, standard error of the mean.

	Individual specimens	Length (µm)	Width (µm)	Intrawall (µm)	Interwall (µm)
		Mean ± SE	Mean ± SE	Mean ± SE	Mean ± SE
Phymatolithon calcareum	FH1E	12.58 ± 0.116 (BCD)	7.19 ± 0.108 (A)	1.25 ± 0.030 (A)	1.70 ± 0.031
	FH2B	12.58 ± 0.120 (BCD)	7.16 ± 0.117 (A)	1.31 ± 0.024 (AB)	1.70 ± 0.036
	FH4B	11.76 ± 0.151(BC)	7.66 ± 0.086 (ABC)	1.53 ± 0.045 (ABC)	1.67 ± 0.031
	FH4D	11.39 ± 0.138 (AB)	7.62 ± 0.086 (AB)	1.52 ± 0.041 (ABC)	1.69 ± 0.036
	FH4E	12.23 ± 0.144 (BCD)	7.98 ± 0.107 (ABC)	1.60 ± 0.035 (ABC)	1.67 ± 0.029
	FH5A	11.27 ± 0.150 (AB)	8.24 ± 0.089 (BC)	1.72 ± 0.043 (C)	1.91 ± 0.036
	FH5E	13.23 ± 0.186 (CD)	8.46 ± 0.104 (C)	1.34 ± 0.037 (AB)	1.66 ± 0.035
	FH6B	9.36 ± 0.215 (A)	7.84 ± 0.159 (ABC)	1.71 ± 0.081(BC)	1.75 ± 0.074
Statistics[a] (*P. calcareum*)		**	**	**	*[b]

	Individual specimens	Mg/ Ca ratio (mol/ mol)
		Mean ± S.E.
Lithothamnion glaciale	OB12	0.172 ± 0.0003
	OB25	0.186 ± 0.0003
	OB32	0.191 ± 0.0004
Statistics[a] (*L. glaciale*)		**

[a]* represents results that have a p-value below 0.05, ** represents results that have a p-value below 0.005 and / represents results that are not statistically significant, letters represent the different statistical groups within each morphological parameter. [b]post hoc test could not distinguish between groups

S2: Finite models showing the differences in stress distribution under compression loading associated to the morphology of the investigated species through time (a, b, c, d, e, f, g). Red colours indicate high stress and blue colours lower stress. Schematic of the boundary conditions where the arrows represent the loads and the black box represents the constraint applied (h). Scale bar: 10 µm. Units: MPa.

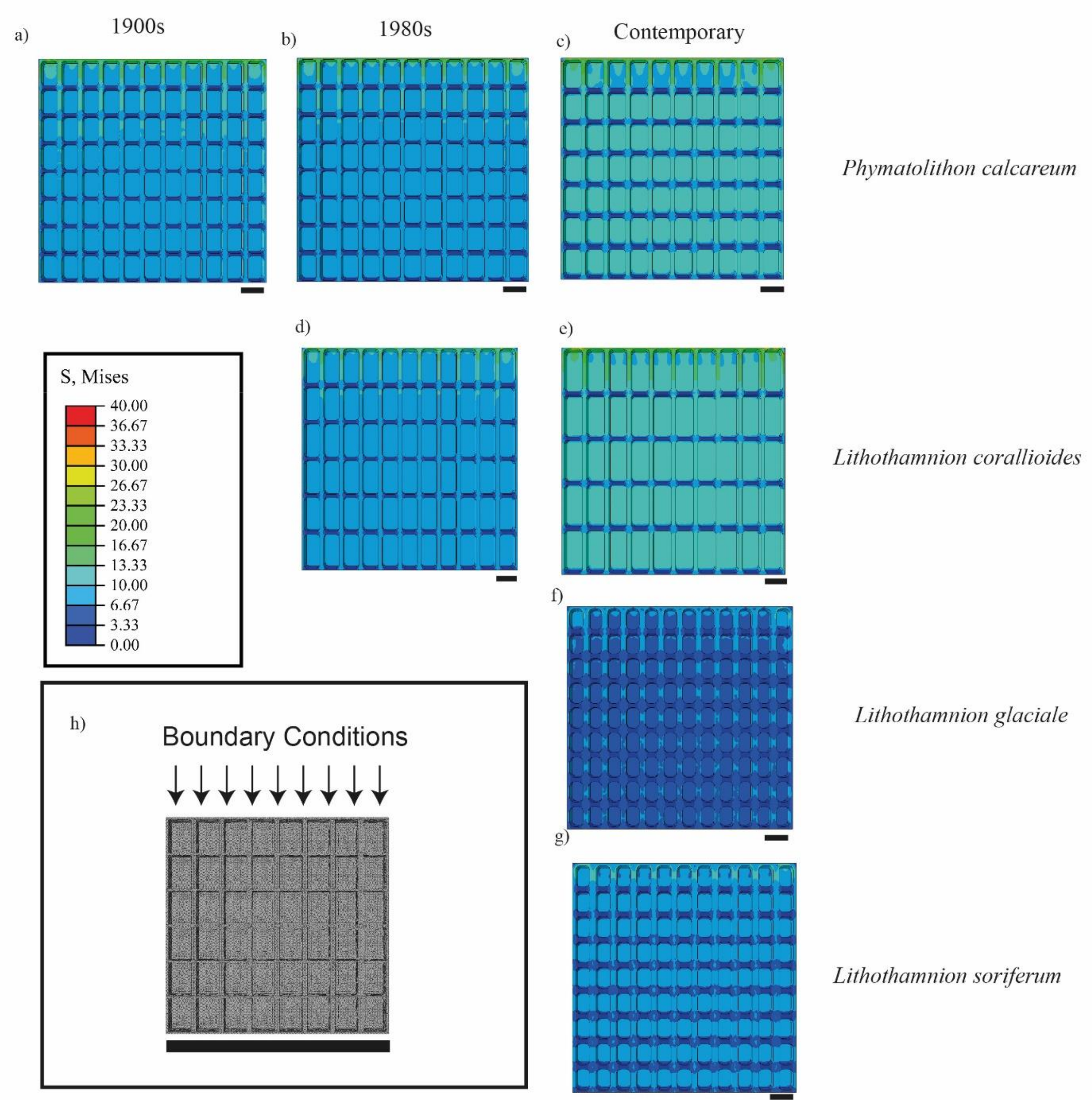

S3: Specimen information for all contemporary and historical specimens: # = number, LC = Loch Creran SAC, Oban, Scotland, UK, St M = St Mawes Bank, Fal & Helford SAC, Falmouth, England, UK; FH = Falmouth Harbour, Fal & Helford SAC, Falmouth, England, UK.

Sample Name	Species	Barcode	Month, Year collected	Location	Time period	Skeletal variability - # of branches	Mineral variability (transects) - # of branches (# of transects per branch)	mineral variability (maps) - number of branches
OB12	*L. erinaceum*	BM001150563	Oct, 2014	LC	Contemporary	3	3 (2)	1
OB25	*L. erinaceum*	BM001150576	Oct, 2014	LC	Contemporary	3	3 (2)	3
OB32	*L. erinaceum*	BM001150583	Oct, 2014	LC	Contemporary	3	3 (2)	3
OB01	*L. glaciale*	BM001150552	Oct, 2014	LC	Contemporary	3	N/A	1
OB07	*L. glaciale*	BM001150558	Oct, 2014	LC	Contemporary	3		1
OB11	*L. glaciale*	BM001150562	Oct, 2014	LC	Contemporary	3		1
OB15	*L. glaciale*	BM001150566	Oct, 2014	LC	Contemporary	3		1
OB16	*L. glaciale*	BM001150567	Oct, 2014	LC	Contemporary	3		1
OB27	*L. glaciale*	BM001150578	Oct, 2014	LC	Contemporary	3		1
FH1A	*L. corallioides*	BM001150587	Jul, 2014	St M	Contemporary	5	N/A	0
FH1C	*L. corallioides*	BM001150589	Jul, 2014	St M	Contemporary	5		1
FH1D	*L. corallioides*	BM001150590	Jul, 2014	St M	Contemporary	3		1
FH2D	*L. corallioides*	BM001150596	Jul, 2014	St M	Contemporary	3		1
FH3C	*L. corallioides*	BM001150599	Jul, 2014	St M	Contemporary	5		1
FH3F	*L. corallioides*	BM001150602	Jul, 2014	St M	Contemporary	3		1
FH3H	*L. corallioides*	BM001150604	Jul, 2014	St M	Contemporary	5		1
FH1E	*P. calcareum*	BM001150591	Jul, 2014	St M	Contemporary	5	N/A	0
FH2B	*P. calcareum*	BM001150594	Jul, 2014	St M	Contemporary	5		1
FH4B	*P. calcareum*	BM001150607	Jul, 2014	St M	Contemporary	5		0
FH4C	*P. calcareum*	BM001150608	Jul, 2014	St M	Contemporary	5		1
FH4D	*P. calcareum*	BM001150609	Jul, 2014	St M	Contemporary	8		1
FH4E	*P. calcareum*	BM001150610	Jul, 2014	St M	Contemporary	5		0
FH5A	*P. calcareum*	BM001150614	Jul, 2014	St M	Contemporary	8		1
FH5E	*P. calcareum*	BM001150615	Jul, 2014	St M	Contemporary	5		1
FH6B	*P. calcareum*	BM001150617	Jul, 2014	St M	Contemporary	5		1
H08	*P. calcareum*	BM000569039	1895	FH	1900s	1	N/A	1
H11	*P. calcareum*	BM000044985	1905	FH	1900s	3		1
H14	*P. calcareum*	BM000044982	1907	FH	1900s	2		1
H01	*P. calcareum*	BM001150619	1976	FH	1980s	3	N/A	1
H17	*P. calcareum*	BM000774728	1985	FH	1980s	2		1
H01 cor	*L. corallioides*	BM001150619	1976	FH	1980s	3	N/A	1
H09	*L. corallioides*	BM000568822	1985	FH	1980s	2		1
Hcor82	*L. corallioides*	n/a	1982	FH	1980s	2		1

S4: Line graphs showing the cumulative average and standard error for each contemporary species.

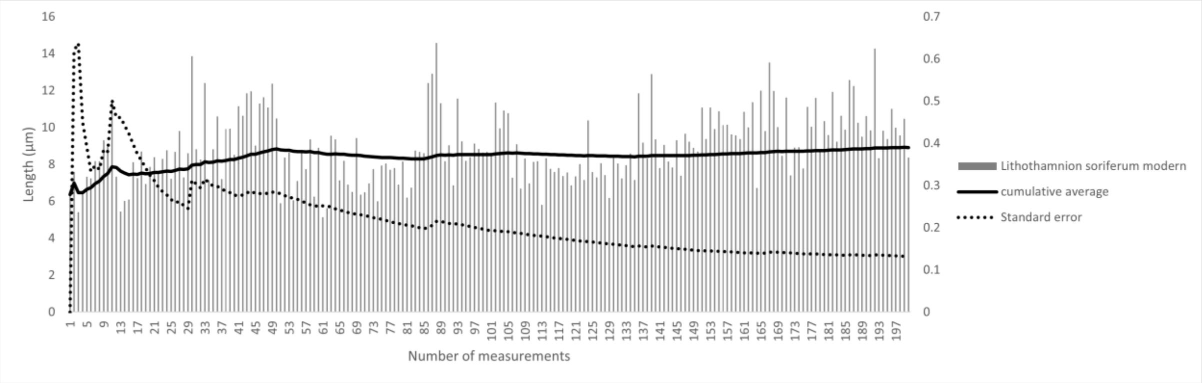

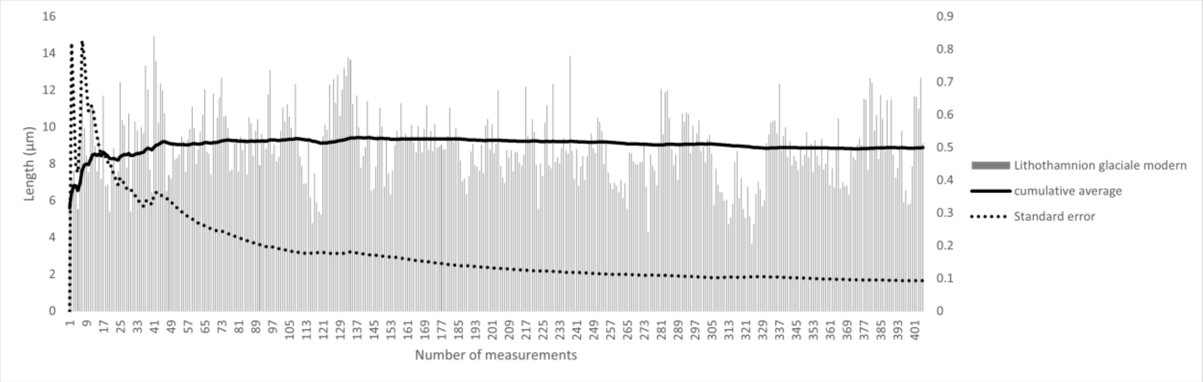

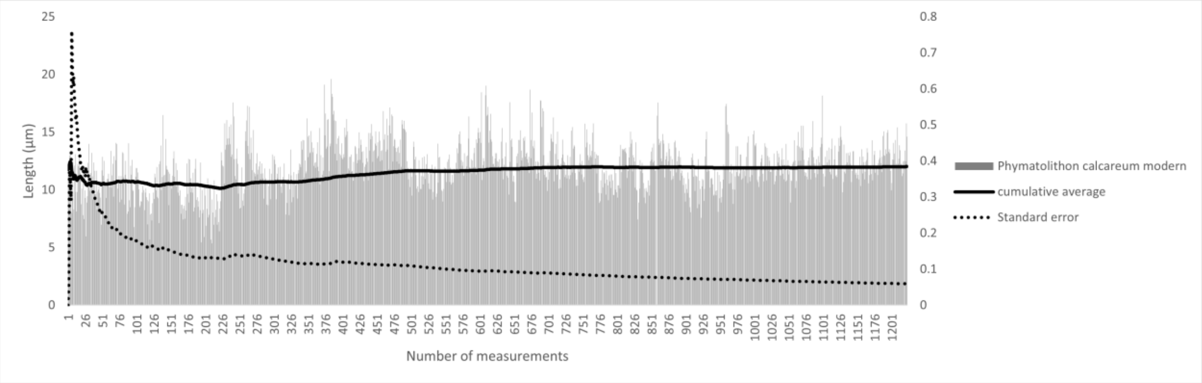

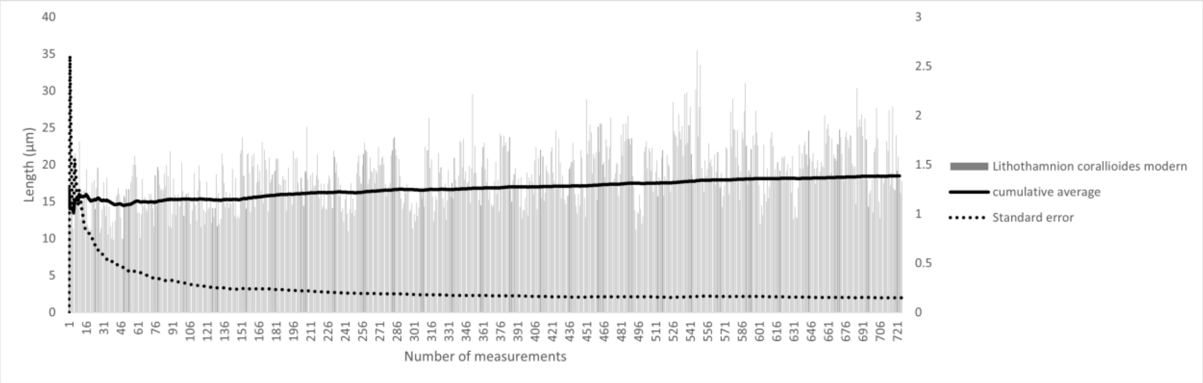

S5: Line graphs showing the cumulative average and standard error for the historical species.

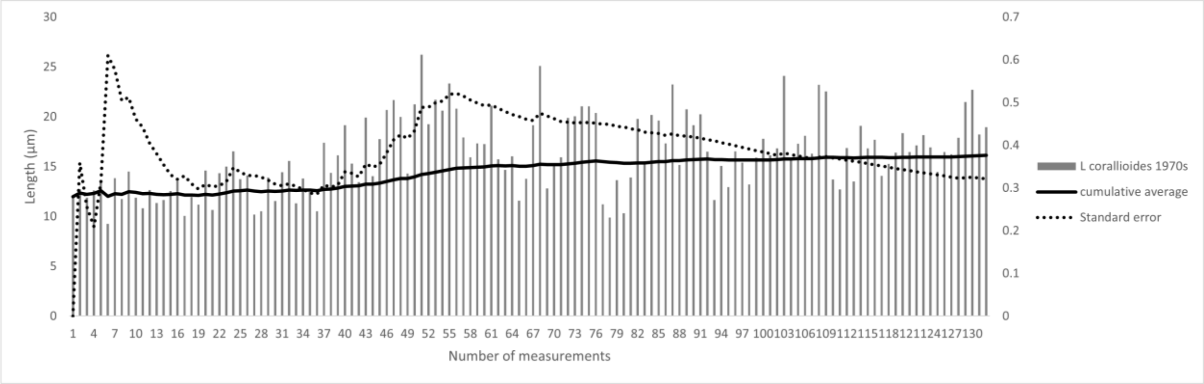

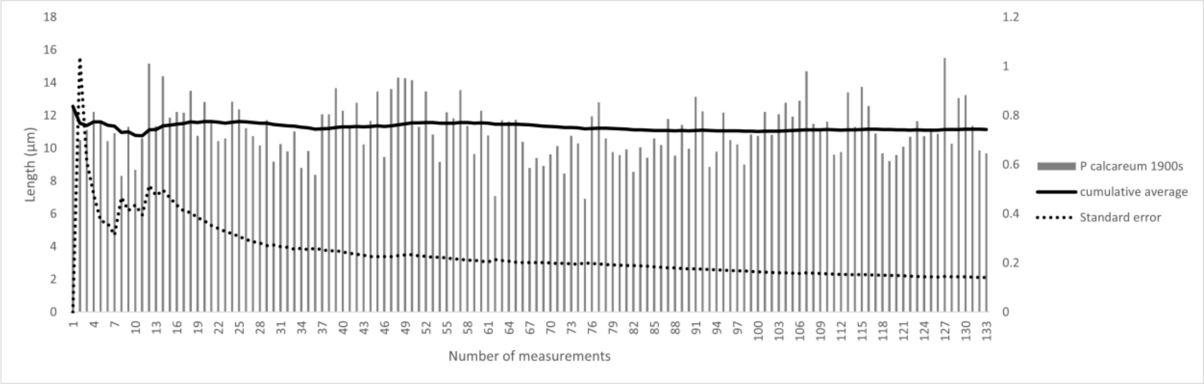

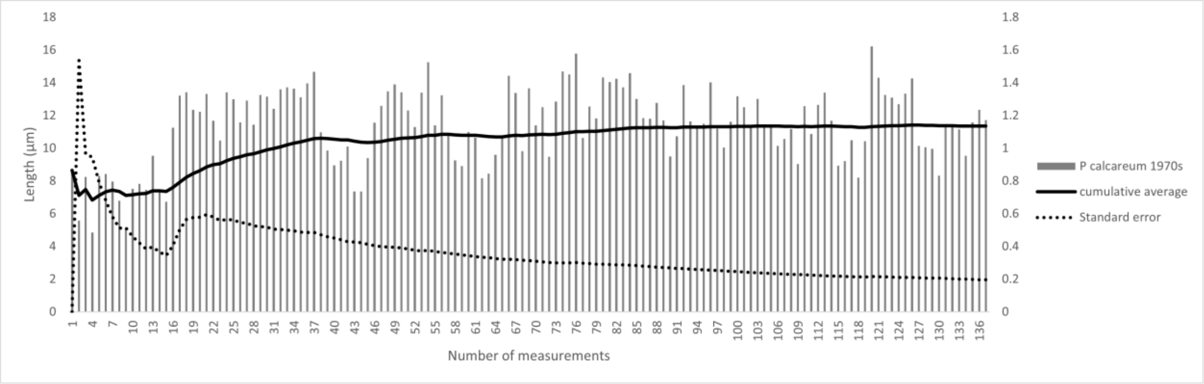

S6: Raman spectrum (grey line) collected from sample Hcor_82. The black dashed line is a fit to the background, and the black dotted line is its upper 95% prediction band; peaks above this line are statistically significant and are shaded in grey. The vertical dashed red and blue lines represent the expected positions of the peaks for pure calcite (i.e., 0 mol% $MgCO_3$) and dolomite (i.e., 50 mol% $MgCO_3$) respectively from Perrin et al. (2016)

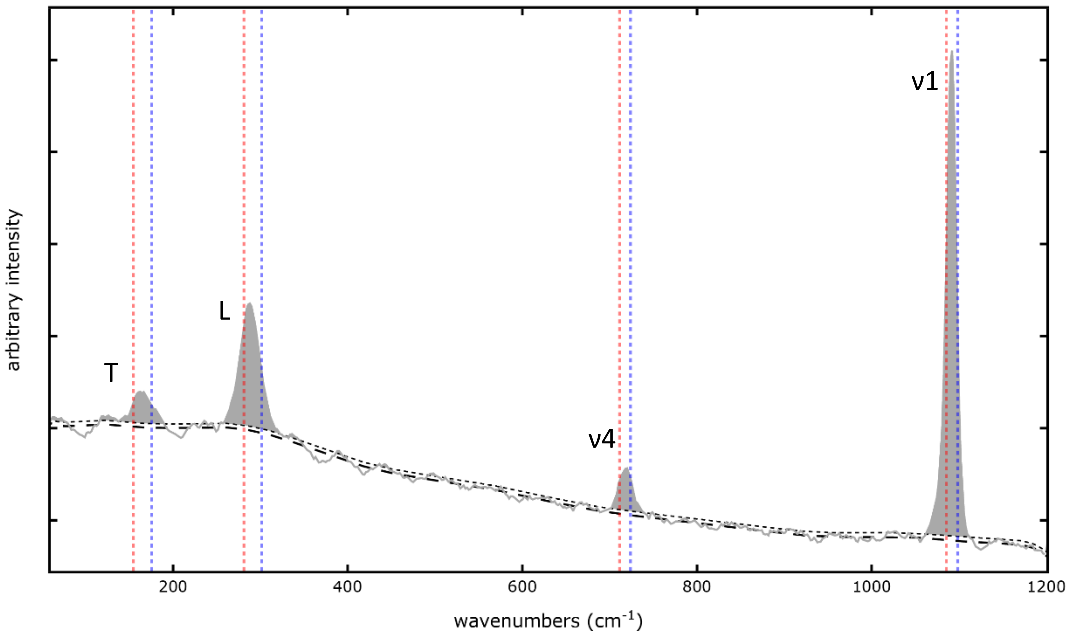

Loading [MathJax]/extensions/MathMenu.js

S7: R code for statistics. Nested Anova, Mixed effects and modified Tukey post hoc test.

Nested Anova

```
library(nlme)
model=lme(A~Sample,random=~1|Branch, data=B, method="REML")

### A = morphological parameter (Length, Width, Intra cell wall, Inter cell wall or Mg/Ca ratio)
### B = database

library(multcompView)
library(emmeans)
library(multcomp)
anova.lme(model, type="sequential", adjustSigma = FALSE)
leastsquare = lsmeans(model, pairwise~Sample, adjust="tukey") ###post hoc Tukey test
cld(leastsquare, alpha=0.05,Letters=letters,adjust="tukey")
hist(residuals(model), col="darkgrey")### check residual histagram for normality
plot(fitted(model), residuals(model))### chekc residuals are unbiased and homoscedastic
```

Mixed Effects model

```
require(lme4)
require(emmeans)
f1<-lmer(A~Species+Time+(1|Individual), B)

### A = morphological parameter (Length, Width, Intra cell wall, Inter cell wall or Mg/Ca ratio)
### B = database

f2<-lmer(A~Time+(1|Individual), B)
anova(f1,f2)
emmeans(f1,list(pairwise~C), adjust="tukey") ### Adjusted Tukey posthoc test

### C = effect (Time or Species)
```
